# On the Element That Kills in Chloroform, and Other Allied Chemicals*Read before the South Wales and Monmouthshire Branch, July 5th.

**Published:** 1871-10

**Authors:** J. D. Brown

**Affiliations:** F.R.C.S. Eng., Haverfordwest


### ON THE ELEMENT THAT KILLS IN CHLOROFORM, AND OTHER ALLIED CHEMICALS.*

By J. D. BROWN, F.R.C.S. Eng., Haverfordwest.

Of all deaths, the quickest are those which follow the total arrest of circulation, where the action of the heart is first stopped. Next in rapidity are those that follow the total arrest of respiration. Death from prussic acid is a fearful example of the first; from drowning and strangling, of the second. By prussic acid, death is instantaneously produced; in drowning and strangling, life is never prolonged beyond three minutes. Death from heart-affection is apparently easily explained; but why die from the exclusion of air? Let us imagine a person in full vigor and life, brought up dead from the water. Not a

* Read before the South Wales and Monmouthshire Branch, July 5th.

finger has touched him; not a drop of his blood has been shed; no poison has polluted his blood; yet he is dead, and three minutes ago he was full of lusty life. Why has he died? His organization is still perfect. The reply, I expect, is, that he died because there was no arterial blood to circulate, oxygen having been excluded long enough to kill. In heart-disease the answer will be, that there was no heart-power to circulate the blood, although it was still arterial and pure. All this is plain enough.

We will now take up chloroform, ether, olefiant gas, and alcohol, with their varieties, in which I shall include all the compounds of carbon, whatever name may represent them, such as chloral, etc. Those chemicals are introduced into blood either as liquids or gases; it matters but little how. In considering their chemistry, we find them richly charged with carbon in subtle solution. The little oxygen, hydrogen, or chlorine, that they contain, simply play the part of solvents to the carbon, and, as far as killing goes, can play no part.

There are two elements brought into play in their application; viz., the introduction of carbon, and the gradual exclusion of oxygen and nitrogen, carbon being retained on the one hand while it is being introduced on the other.

By excluding air, we can arrive at death without the aid of any other agent. By introducing carbon, we can reach the same point without calling upon strangling to help us. This is precisely what we accomplish in giving any of those subtle solutions of carbon, either as liquids or as gases. We gradually exclude the air and as gradually introduce the compound till we reach a given point, at which the blood is fully saturated with it, the agency of those two powers being put into force. It matters but little how we effect our aim; all that is wanted to complete our object is carbon, in sufficient quantity to overpower the brain and its functions. Drowning and strangling would answer our purpose quite as well as any of the chemicals in use, only we cannot as yet manage such dangerous agents with as much safety as we could wish. Moreover, they are painful modes, and never likely to be employed.

Of all our agents the pleasantest is chloroform, which, whilst killing, indulges the victim with the pleasant delusions of a happier life, and engages the respiratory organs at the same time in their usual work; thus throwing the whole living system off its guard, which, although apparantly rejoicing in life, is in reality slowly dying. This is truly a happy death. The introduction of any of these compounds through the stomach produces the same effects.

Alcohol and chloral are well known agents, capable of intoxicating and even killing by introducing carbon too suddenly and in excess. To remove their effect, oxygen must be called to our aid, which, gradually combining with the elements of the intoxicating substance, at last expels the overcharge in the form of carbonic acid, water, etc.

Having exhausted the compounds of carbon, and proved their terrible powers of stupefying and killing, we shall take up those possessed of the same power, and which yet contain no carbon. This is a paradox. Here, you will say, a difficulty arises, a barrier rears its head, over which one will not very easily pass. I can see none. We have looked on the fearful rapidity with which drowning and strangling kill. In what does their power consist? Simply in excluding air. We give a gas, such as nitrous oxide, nitrogen, or any other respirable gas—even hydrogen, and we will not even despise carbonic acid—the results are precisely the same; they kill with a rapidity that might be measured by the quantity of air allowed to mix with the gas used—in fact, we amuse the lungs and throw them off their guard whilst preventing the oxidation of the circulating carbon This is much more pleasant than drowning, but equally fatal.

In nitrous oxide and carbonic acid we have compounds satisfied with their chemicals; therefore, neither the carbon nor oxygen is brought into play. They simply exclude atmospheric oxygen, thus leaving the "killing" to the accumulating carbon. Flame, in ceasing to live in carbonic acid, simply dies for want of oxygen, not because carbonic acid is present, which, if it were to decompose, would yield sufficient oxygen to maintain combustion. An animal perishes in carbonic acid for the same reason. Chloroform has no advantage over carbonic acid except that its carbon is probably not free: I say probably, as there is no certainty.

*Cause of Death.*—It is not the detention of carbonic acid that kills, but the presence of carbon in some peculiar state, and unoxidised. The small quantity of carbonic acid which the last inspiration creates, even if taken up by the blood and not imprisoned in the air-cells, would be too little to kill, granting it had the power. Death is really caused by paralysis of the heart. In every instance of death in rabbits, the circulation first failed, beginning in the ears; danger was always plainly visible there; and when chloroform was further given to the complete arrest of circulation, death always ensued. On *post mortem* examination, the heart was found to beat, but not with sufficient vigor to empty its chambers. No blood seemed to pass through the lungs to the left chambers. There was an idle action, simply from the fact that the blood went home to die—viz., to the venous system, whose headquarters is the right side of the heart and lungs. The last inspiration, shallow and superficial as it is, may be taken as a measure of the last stream poured out from the left ventricle to complete the circle for the last time, and then to stop forever. The heart fails, because highly carbonised blood cannot maintain the vigor of the nervous system. The moment circulation ceases, respiration comes to a dead stand; to re-commence the old work, circulation must take the initiative; respiration follows. This is the ordained order. With stagnant blood there can be no breathing. A newly born child breathes because his blood circulates: no human power could command a single breath had that function entirely ceased.

We now can understand why artificial respiration so often fails: in truth, we begin where we ought to end. Blood can only be oxidised in circulation; no amount of oxygen could tell upon the small quantity stagnant in the lungs—a proportion infinitely too small to rouse the nervous system even were it to move forwards. The first step toward life the heart must make; the second the lungs will take.

*Indications of death* are pallor and white lips; circulation has failed or is failing; a flushed or dark countenance is safe. Breathing you need not notice; it cannot go wrong. As long as the circuluation is right, the first step to death is made by the circulation, the second by the respiration.

How to avert the impending death is the next question. There may still be a little arterial blood in the lungs and left side of the heart. There is just one chance: pour it into the brain by hanging the head down. If the brain respond, all is safe; the heart resumes its labors and the lungs answer to the call; and, unless we succeed in rousing the brain by the little arterial blood which the vessels may still hold, the case is hopeless.

It is impossible to manufacture arterial blood without oxygen; and it is not possible to get air to tell upon the whole volume of blood unless we can insure its circulation; and it cannot circulate without the heart's permission, and the heart will not act till the nervous system is able to command it, and until the blood gets rid of its overcharge of carbon. Artificial respiration is useless, and is a loss of time till the circulation resumes its labors.

Dragging out the tongue is an idle occupation; the mouth is not the passage for air. The glottis cannot be closed by a dead tongue and retracted epiglottis. The only instances of its closure are attended by congestion of face and brain, and not by pallor and empty vessels. Observe the face in epilepsy, in drowning and strangling, convulsions, stammering and choking from spasms, or by intruding morsels or liquids. The glottis is never closed, only whilst swallowing; no amount of stupor shuts it, and even in death it is always open; in fact, that is its normal condition, excepting in those diseases just named.

To me it is a question yet to be decided whether we could not shorten convulsions by keeping open the glottis if means could be devised. Stammering is always relieved by preventing its spasmodic closure, by keeping a marble in the mouth, by drawling, or by intonation of speech, as singing or reading poetry.

*Summary.*—No time should be lost in artificial respiration, dragging out the tongue, etc.; but the brain should be roused by hanging down the head. There can be no harm in artificial respiration; it may be carried on at the same time. The cases appended all prove the value of this plan. It is also applicable to drowning, and to all the other causes of death just enumerated.

I append a few cases illustrative of the treatment recommended.

Case I. A stout woman, aged 50, had her breast removed for cancer. Whilst we were securing the arteries, the circulation suddenly stopped. She was to all appearance dead—no pulse, no breathing, no color, but a ghastly white. At this critical moment I struck away all the pillows, and, with aid, laid her on the floor, her head lower than her body. Her lips became red, and in time she breathed, and by degrees recovered. There was no attempt at artificial respiration, nor was the tongue drawn out.

Case II. A young and healthy peasant girl had her leg amputated, to improve a bad stump left by mortification of her foot and lower third of leg, caused by typhus fever. We were dressing it, when suddenly bleeding ceased. She had been neglected by the chloroformist, who left the handkerchief too near to her nostrils. She was breathless, pulseless, and pallid. Her head was raised on a block and pillow, for the operation, which I struck boldly away; her head fell with an audible noise on the table; at that moment the lips reddened, the face flushed, she breathed, and was safe.

Case III. A lady, young and healthy, took chloroform to have a tooth extracted. Pallor at last followed, accompanied with the usual signs of death from chloroform. This was early in my experience of chloroform, and I was much frightened. She was laid down, her head lowered, and by and bye her lips showed signs of life, breathing returned, and she was saved.

Case IV. A man aged about 56, delicate, and worn by long suffering, had his thigh amputated. Whilst the wound was being dressed circulation ceased, and he was apparently dead. His head was hung down over the table, and he soon recovered like all the others.

Case V. An infant, whilst under chloroform for the removal of a nævus, became alarmingly pale, and apparently died. So much had been said about artificial respiration and dragging the tongue out, that we tried it, and thereby lost time; it failed entirely; we then resorted to our old plan. The child was literally hung upside down, till the lips grew red, and all was safe.—*Brit. Med. Journal.*

---

Cats and Dogs are proved to be insusceptible of the vaccine disease.

